# Strength Properties and Microscopic Experimental Study of Modified Sawdust Based on Solid Waste Synergistic Utilization

**DOI:** 10.3390/ma17235808

**Published:** 2024-11-27

**Authors:** Yu Cheng, Na Jiang, Wentong Wang, Lu Jin

**Affiliations:** College of Transportation, Shandong University of Science and Technology, Qingdao 266510, China; skd996718@sdust.edu.cn (Y.C.); jiangna@sdust.edu.cn (N.J.); skd991767@sdust.edu.cn (L.J.)

**Keywords:** modified sawdust, unconfined compressive strength, SEM-EDS, wet and dry cycles, expansion and contraction characteristic

## Abstract

Sawdust is the cutting tailings produced during stone processing, which is difficult to deal with and has a huge stock. Therefore, it is particularly important to enhance the comprehensive utilization of sawdust. The aim of this study was to synergistically utilize sawdust with other industrial wastes (fly ash, silt, and red mud), add cement as a curing agent to prepare modified sawdust, and analyze its performance through an unconfined compressive strength test, dry and wet cycle tests, and SEM. The results showed that the compressive strength of modified sawdust with different solid waste dosages was more than 2.5 MPa after 7 days of maintenance, the strength was basically more than 4 MPa after 28 days of maintenance, and 8% solid waste dosage had the best effect. In addition, the modified saw mud with 8% fly-ash dosage had superior wet and dry cycle resistance, with expansion and shrinkage lower than 0.5% and good stability. This study provides a new idea for the synergistic utilization of saw mud and other solid wastes, and it is recommended to consider 8% solid waste dosage to optimize the performance in practical applications.

## 1. Introduction

The cutting tailings produced during the processing of stone, such as granite stone powder or marble powder, are the main source of sawdust [1]. Sawdust is a mixture of stone powder and water with characteristics such as fine texture, high water content, and high viscosity, which make it difficult to treat and utilize directly. Currently, only centralized recycling and stockpiling can be done [2], but large stockpiling can lead to serious environmental pollution problems [3,4]. Therefore, enhancing the utilization of sawdust is an important step towards the realization of green industry. In engineering applications (e.g., roadbed filling, etc.), sawdust is required to have a certain level of strength and stability. This can be achieved by incorporating composite materials so that they meet the technical requirements for engineering applications.

There have been some developments regarding the use of composites in roadbed filling, such as evaluating the impact of mixing plastic wastes in asphalt [5] and research on the reinforcing properties of fibers in asphalt mixtures, especially polyacrylonitrile fibers [6,7]. The feasibility of sawdust as a roadbed fill has also been explored [8]. For example, Vikas et al. [9] evaluated the effects of different concentrations of sawdust on the index and engineering properties of black cotton soil, and the results showed that sawdust accounting for 30% of the dry weight of the soil could improve the geotechnical properties of expansive soils and could be used for roadbeds of low-volume roads. Amani et al. [10] investigated the effects of replacing natural fine aggregates by using marble powder and granite powder on the main mechanical properties of concrete pavements. The results showed that the combination of the two had better mechanical properties. Zhao et al. [11] found that granite powder mixed with industrial by-products such as steel slag could improve the water damage resistance and spalling resistance of asphalt mixtures.

Fly ash is a low-cost industrial by-product. Because of its volcanic nature, the active SiO_2_ and Al_2_O_3_ contained therein can react with Ca(OH)_2_ in the cement to generate cementitious substances such as hydrated calcium silicate, which can improve the durability of concrete [12,13]. Granite powder, on the other hand, improves densification and reduces permeability [14,15]. For example, Jain et al. [16] analyzed the mechanical and microstructural properties of self-compacting concrete consisting of fly-ash blends with granite wastes, and Lieberman et al. [17] evaluated the potential of granite wastes produced from quarries in Galicia, Spain, blended with fly ash as a partial replacement of natural sand in concrete. Furthermore, Chajec et al. [18] found that partial replacement of cement in concrete by the addition of granite powder and fly ash can help to reduce CO_2_ emissions into the atmosphere associated with cement production. The utilization of silt is relatively rare in road engineering, but it has been shown that the mechanical properties of silt can be improved through the use of binding agents such as cement, lime, and fly ash, and its strength can meet the requirements of road subgrade, making it suitable for use as a road construction material [19,20]. In addition, Xiao et al. [21] proposed the concept of mixing granite powder as an admixture into cemented coastal silt to solve the problem of recycling granite powder and to improve the strength of cemented coastal powder. Red mud is a by-product of the bauxite refining process, which is highly alkaline and environmentally risky [22]. However, the utilization of red mud in road subbases and asphalt mixtures shows feasibility, with good unconfined compressive strength, frost resistance, and durability [23,24]. Dong et al. [25] prepared closed-cell foamed ceramics by the powder sintering method using granite waste and red mud as raw materials and SiC as a blowing agent to realize the comprehensive utilization of granite waste and red mud. Muraleedharan et al. [26] reviewed and summarized the studies on the production of geopolymer binders and mortars using red mud and granite waste powder. Chakkor et al. [27] prepared samples of metakaolin and red mud base polymers from limestone powder, marble powder, and basalt powder and compared the mechanical properties thereof. The utilization of fly ash, sludge, and red mud is an effective method of industrial solid waste reduction and comprehensive utilization on a large scale, which helps to reduce pollution to the environment and realize the double benefits of environmental protection and engineering construction.

In fact, the comprehensive utilization of sawdust has been explored in a number of ways, including the study of the properties of concrete produced from sawdust [28,29], the characterization and evaluation of cementitious composites [30,31], and the sustainable development of sawdust as a substitute [32,33]. However, different industrial wastes have different effects on the comprehensive utilization of saw mud, and a change in the admixture amount also has certain effects on the modified saw mud. Therefore, this study breaks through the limitation of the traditional single-solid-waste blending study by mixing three different industrial wastes, namely fly ash, silt, and red mud, with saw mud separately to prepare modified saw mud. In this study, the effects of different industrial wastes and different blending amounts on the strength of modified saw mud were investigated comparatively through an unconfined compressive strength test, expansion and contraction tests, dry and wet cycle tests, and an SEM microscopic test. This kind of solid waste utilization not only enriches the research field of solid waste resource utilization but provides new material choices and construction methods for highway roadbed filling projects and promotes the sustainable development of industrial solid waste recycling.

## 2. Test Materials and Methods

In this study, modified sawdust was prepared using cement as a curing agent and sawdust, fly ash, silt, and red mud as raw materials. The strength properties of the modified sawdust were tested by an unconfined compressive strength test, wet and dry cycle tests, expansion and contraction tests, and a microscopic test. The chemical composition of the raw materials was analyzed by an X-ray fluorescence spectrometer (XRF), model ZSX Primus IV from Rigaku Corporation, Tokyo, Japan, with a goniometer accuracy of ±0.0001° and a scanning rate of 2400°/min. The mineralogical composition was measured by an X-ray diffractometer model D/Max2500PC with an accuracy of ±0.0001° and a scanning rate of 2400°/min. The goniometer radius was ≥200 mm, the scanning range was 5–50°, and the scanning speed was 5°/min. The liquid–plastic limits of each material were determined by an LP-100 D liquid–plastic limit meter with a drop weight of 100 g.

### 2.1. Raw Materials

#### 2.1.1. Sawing Mud

**Figure 1:**
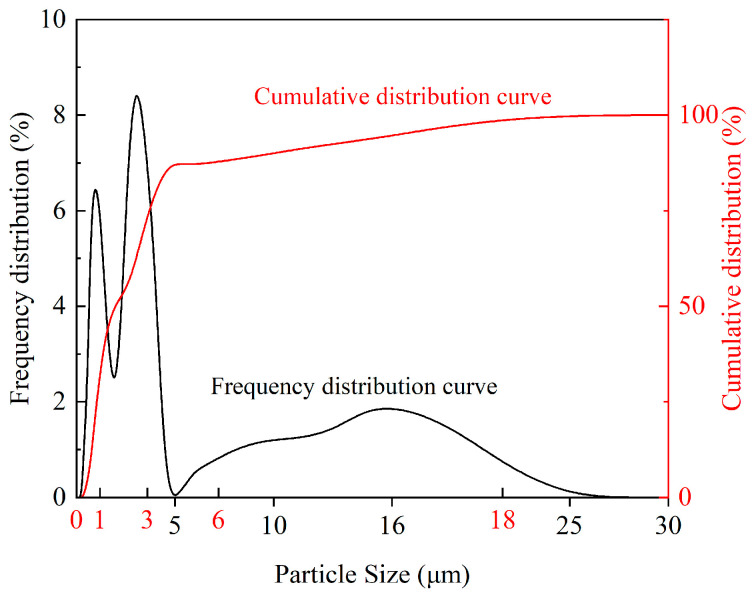
Sawdust particle size distribution curve.

The sawdust used in this study was sampled from the granite quarry base in Wulian County, Rizhao City. Its maximum dry density was 1640 kg/m^3^, and the optimum moisture content was 18%. The particle size distribution curve of the sawdust is shown in Figure 1. The main particle size distribution was below 10 μm, accounting for about 90% of the total; the particles below 0.6 μm accounted for 10%, and the median particle size D50 was 1.64 μm. This narrow distribution of particle sizes led to better performance in use. The chemical composition is shown in Table 1; the main chemical component was SiO_2_.

**Figure 2:**
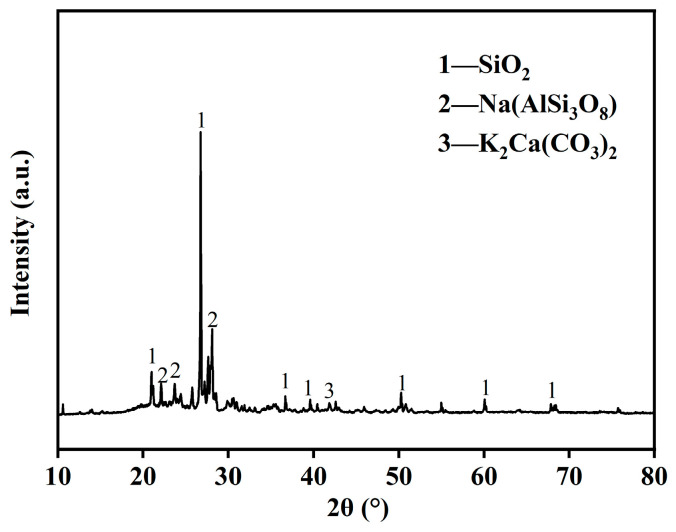
XRD pattern of saw mud.

The XRD pattern of the saw mud is shown in Figure 2, and the main mineral components were quartz, plagioclase feldspar, orthoclase feldspar, and a small amount of smectite, with a relatively high content of potassium in the chemical composition. Sawdust is chemically stable, with strong or very strong resistance to water and chemical weathering. Among the mineral components, quartz is the strongest, so it is also the main mineral component of the powder grains and can even be a coarse clay grain, while feldspar mica minerals gradually weather into secondary minerals when the grain size is very small.

#### 2.1.2. Cement

The cement selected for this experiment was Qufu Zhonglian benchmark cement, the hydration reaction products of which can enhance the connection strength between soil particles and improve the crack resistance of modified saw mud. Therefore, it was used as a curing agent in the modified saw mud. Its basic physical properties are shown in Table 2, and its main mineral components were tricalcium silicate (3CaO–SiO_2_), dicalcium silicate (2CaO–SiO_2_), and tricalcium aluminate (3CaO–Al_2_O_3_); the chemical composition is listed in Table 1.

#### 2.1.3. Fly Ash, Silt, and Red Mud

The fly ash used in the test was sampled from Pingyin, Jinan, with a density of 2132 kg/m^3^ and a specific surface area of 1.82 m^2^/kg.

Silt is treated wastewater discharge deposition formed by biochemical action. It has low mechanical strength and compression, a maximum dry density of 1300 kg/m^3^, optimal water content of 16.4%, and a main mineral composition of quartz and feldspar.

Red mud is used the aluminum industry to extract alumina industrial slag. That used in the present study was sampled in Binzhou, Shandong, North Sea, with a maximum dry density of 1820 kg/m^3^, a best moisture content of 24.1%, and a main mineral composition of aragonite and calcite, which made up 60% to 65% of the overall mineral content.

The liquid–plastic limits of these materials are shown in Table 3.

### 2.2. Experimental Design

In the present study, the blending design of modified saw mud was investigated on the basis of saw mud. In order to determine the blending ratio of cement, the prepared specimens were pretested by a percussion test to determine the maximum dry density and optimum moisture content. Finally, the cement content was fixed at 6% based on the test results. Based on the results of the unconfined compressive strength pretest (see Table 4), the doping range of the three materials from 0 to 10% already met the strength requirements of medium- and light-traffic base and subbase of secondary and lower secondary highways in the Chinese standard Technical Rules for Construction of Highway Pavement Base Levels (JTG/T F20-2015) [34]. Moreover, the unconfined compressive strength was basically unchanged when 15% and 20% of silt and red mud were mixed externally, compared with 10%. Considering the cost, the dosages of fly ash, silt, and red mud were determined as 0%, 3%, 6%, 8%, and 10%. Three parallel groups were set up for each modified saw mud specimen. The letter M was used for saw mud, C for cement, F for fly ash, S for silt, R for red mud, and the number for solid waste admixture. The specific formulations are listed in Table 5.

### 2.3. Sample Preparation

**Figure 3:**
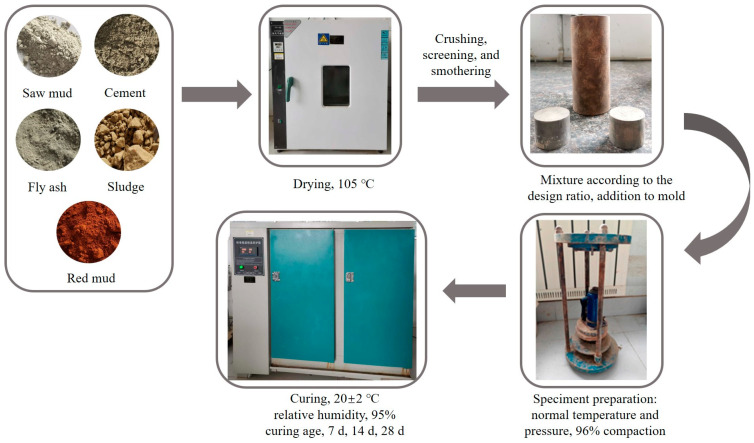
Sample preparation process.

The raw materials were sawdust, cement, fly ash, silt, and red mud in 105 °C oven drying, crushing, and 100-mesh sieve screening. Materials were smothered for a day and night, and then, according to the design ratio, were mixed with the screened solid waste admixture, stirring and mixing evenly, and finally put into a mold at a compaction degree of 96% to prepare a specimen, which was put into a maintenance box for maintenance. The specific flow chart is shown in Figure 3 below.

### 2.4. Test Methods

To study the strength properties and expansion and contraction characteristics of modified sawdust, an unconfined compressive strength test and expansion and contraction tests were conducted on modified sawdust with different types and contents of solid waste admixtures as well as under dry and wet cycles. Microscopic observation and analysis of the specimens were also carried out.

(1) Unconfined compressive strength test

This test method referred to the Chinese standard “Test Procedure for Stabilized Material of Inorganic Binding Material for Highway Engineering” (JTG 3441-2024) [35]. The specimen was a cylinder with a diameter of 39.1 mm and a height of 80 mm. The prepared specimen was sealed in a Ziplock bag and placed in a curing room with a constant temperature of 20 ± 2 °C and a relative humidity of 95%. The test speed was set to 0.36 m/s. The model was cylindrical, and the diameter, 39.1, was entered to record the maximum force value and the compressive strength at this time.

(2) Expansion and contraction tests

This test method adopted the Chinese standard “Technical Code for Construction in Expansive Soil Areas” (GB50112-2013) [36]. Ring-knife pieces were prepared with areas of 3000 mm^2^ and heights of 20 mm. The time and mixing ratios for preparing the modified saw mud ring-knife specimens were recorded and labeled. The specimens were sealed in plastic bags and placed in a constant temperature of 20 ± 2 °C and relative humidity of 95% in a maintenance box 1 d after the expansion and contraction tests.

Because of the long test period, weather changes, and the temperature difference between day and night, this paper set the expansion and contraction tests to be carried out in the oven, setting the temperature at 26 °C. Before the shrinkage test, the modified sawdust specimens were not treated with water immersion. A modified sawdust specimen was taken out with a sampler and put onto a porous plate. A percentage meter was put into a shrinkage meter, fixed, adjusted to zero, and put into the oven for a shrinkage test. The expansion test was carried out without removing the specimen directly. The specimen was put into the shrinkage meter, the base was screwed on, and the percentage meter was adjusted to zero. Water was then poured into the specimen above the height of the bottom or flush with the moisture, and the shrinkage meter, water, and specimen were put into the oven for the test.

(3) Dry and wet cycle test

This test adopted the Chinese standard “Standard of Test Methods for Long-term Performance and Durability of Concrete” (GB/T50082-2024) [37]. After 28 d of specimen curing, the specimen was labeled with a serial number and weighed for mass, and the wet and dry cycle was started. The specimen was into the oven at 30 °C, baked for 16 h, removed for weighing, and then put into an electric constant-water-temperature box. Water was added to the box to cover the surface of the specimen. After 8 h, the specimen was removed, dried, and weighed. A cycle of 24 h, after the end of the cycle, took 3 to 4 specimens to test the unconfined compressive strength. The operation cycle was repeated 7 times.

In the same steps as the above expansion and contraction test, the modified sawdust specimen, after 1 d of maintenance, was taken out from the maintenance box with a sampler, and the shrinkage meter, together with the specimen, was put into the oven at 26 °C for 16 h to simulate the process of demoisturization. The mass was weighed at intervals of 2 h, 4 h, 6 h, 8 h, 12 h, and 16 h. At the end of the test, the expansion test was carried out for 8 h and counted at intervals of 5 min, 10 min, 30 min, 60 min, 120 min, 180 min, 240 min, 360 min, and 480 min. This time was a cycle, and a total of 7 cycles were carried out, with a cycle period of 1 d.

(4) SEM-EDS test

Tests were carried out with a field emission scanning electron microscope (FESEM), model Apreo S HiVac from FEI, Hillsboro, OR, USA, with an electron beam current of 1 pA-400 nA and an accelerating voltage of 200 V–30 kV. The observed samples were naturally crushed and formed as thin flakes of about 5 mm. The micromorphology of MC, MCF-8, MCS-8, and MCR-8 specimens cured for 28 d and after 7 cycles of wet and dry cycling were observed at 8000×, and two points were selected for elemental composition analysis.

## 3. Results and Discussion

### 3.1. Strength Performance Analysis of Modified Saw Muds

#### 3.1.1. Comparison of Strengths After Different Numbers of Maintenance Days Under the Same Mixing Ratio

**Figure 4:**
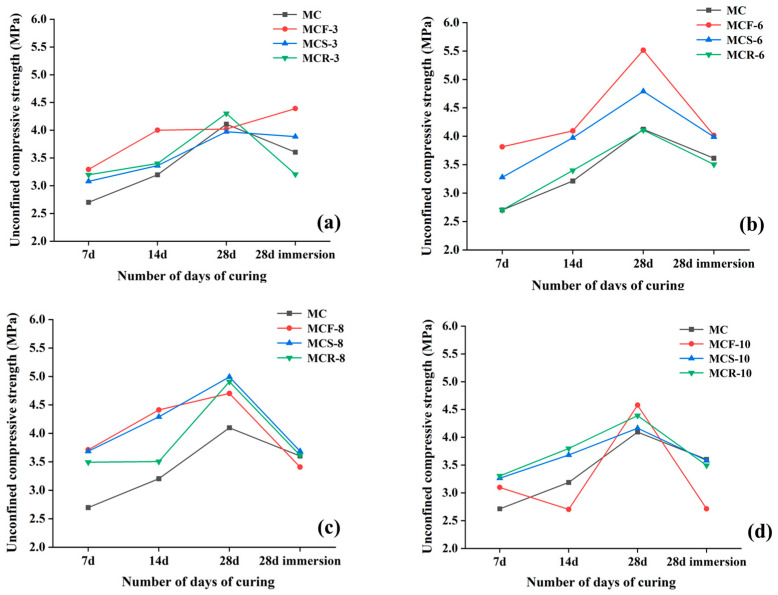
Comparison of the unconfined compressive strengths of modified saw mud with different numbers of days of curing: (**a**) 3% solid waste doping; (**b**) 6% solid waste doping; (**c**) 8% solid waste doping; (**d**) 10% solid waste doping.

Figure 4 shows a comparison of the trends of the unconfined compressive strengths of the modified sawdusts at different days of curing, and as a whole, it can be seen that the unconfined compressive strengths of the modified sawdusts basically increased with increasing numbers of days of curing, but the strengths decreased after immersion in water. This fit with the relationship between the number of days of curing and strength derived from previous studies, where the number of days of curing was directly proportional to the compressive strength of the material. Specifically, the strengths of all modified saw muds peaked after 28 d of curing, and MCF-6 reached 5.5 MPa. The MCR-6 curves basically followed the same direction as the MC curves, suggesting that the synergistic effect of saw mud and 6% red mud was not obvious. The strength curves of MCF-10 fluctuated more, and the curves of MCS-10 and MCR-10 were in line with the overall trend. Together, these results reveal the changes in the performance of modified sawdust and the differences in its synergistic utilization effects under different numbers of maintenance days.

#### 3.1.2. Comparison of Strengths of Different Mixing Ratios Under the Same Number of Maintenance Days

**Figure 5:**
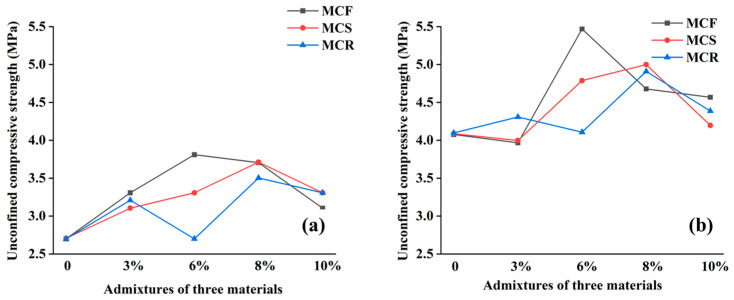
Comparison of the unconfined compressive strength of modified saw mud with different mixing ratios: (**a**) after 7 d of maintenance; (**b**) after 28 d of maintenance.

Figure 5 shows in detail a comparison of the unconfined compressive strengths of modified sawdusts after 7 and 28 days of maintenance. Overall, the compressive strengths of the modified sawdusts showed an increasing and then decreasing trend with increasing dosages. Specifically, as shown in Figure 5a, the strengths of the modified sawdusts after 7 days of curing ranged from 2.5 MPa to 4.0 MPa, and MCF and MCS reached their peak strengths at 6% and 8% dosages, respectively, and then decreased with increasing dosages. As shown in Figure 5b, the strengths of the modified sawdusts maintained for 28 days ranged from 4.0 MPa to 6.0 MPa, with a clear intertwining among the curves. The compressive strength of MCF was significantly higher than the peaks of MCS and MCR at a dosage of 6% and reached 5.5 MPa. These data clearly reveal the effects of different dosages on the compressive strength and performance of modified sawdust.

#### 3.1.3. Comparison of Strengths Before and After Water Immersion

**Figure 6:**
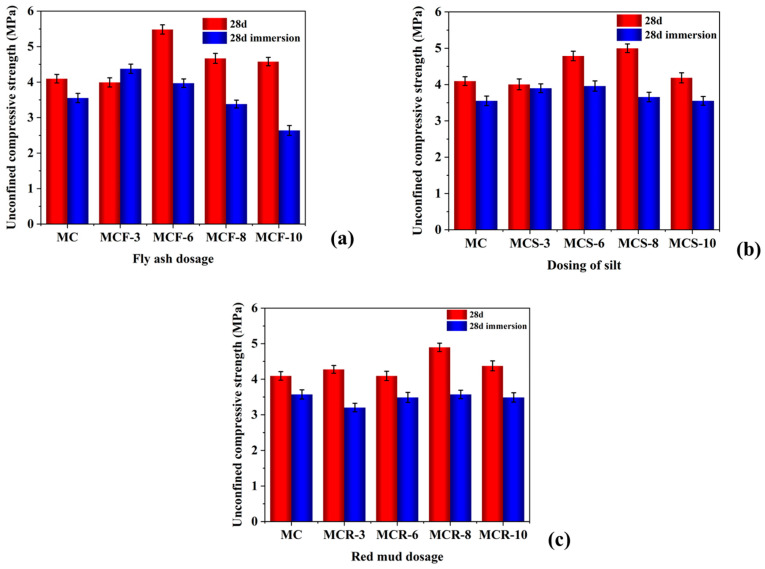
Comparison of unconfined compressive strengths of modified saw mud before and after water immersion: (**a**) fly ash; (**b**) silt; (**c**) red mud.

To analyze the unconfined compressive strength of modified saw mud before and after water immersion, the modifications, after 28 d and 28 d (1 d of water immersion), were tested for compressive strength (Figure 6). From Figure 6a, it can be seen that except for MCF-3, of which the compressive strength increased after water immersion, the compressive strengths of the modified saw muds decreased after water immersion, and the decrease was most pronounced when the dosage was 10%. As can be seen from Figure 6b, the unconfined compressive strengths of MCS after water immersion were all lower than those of MCS without water immersion. However, when externally doped with silt solid waste synergistic utilization, the loss of strength when immersed in water was much smaller than for saw mud externally doped with fly ash. This was because the silt contains some viscous substances with high viscosity, which can play a certain bonding role, improve the cohesion of sawdust and the overall compressive strength. In addition, the hydration reaction of cement releases Ca^+^ ions, and these ions are exchanged with free Na^+^ ions and Ca^+^ ions in the silt to enhance the adsorption between the particles, thus increasing the strength. Silt is mainly carbonates and silicates, which form calcium silicate, sodium silicate, etc. with the oxides in the sawdust, which is mainly silicon oxide, filling the pores and increasing the strength of the composite. As can be seen from Figure 6c, the unconfined compressive strengths of the composites decreased after immersion in water, was stable, and did not change much with increasing dosages of red mud.

**Figure 7:**
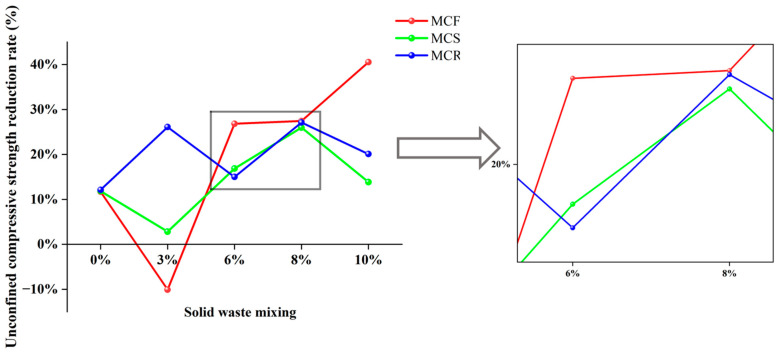
Comparison of losses of unconfined compressive strength before and after water immersion of modified saw muds.

Figure 7 shows the depreciation curves of the unconfined compressive strengths of the modified sawdusts before and after water immersion. From the figure, it can be seen that with increasing fly ash doping, the discount rate of the unconfined compressive strength of modified sawdust after water immersion increased gradually. When the dosage of fly ash was 3%, the discount rate was about −10%, which indicates that the unconfined compressive strength after water immersion was greater than that without water immersion. This may have been due to water immersion promoting the hydration reaction of cement. However, when the fly ash dosage was 10%, the discount rate reached 41%. This suggests that a suitable level of fly ash incorporation contributed to the strength of the modified sawdust and corroborates the study by Akbulut et al. [12] on the reinforcing properties of fly ash. When silt was externally doped, the loss of unconfined compressive strength of specimens after immersion in water decreased and then increased with increasing silt doping and reached a maximum of 25% when the silt doping was 8%. When specimens were mixed with red mud, the loss of unconfined compressive strength was more obvious, and the loss rate of the specimens was between 10% and 25%.

### 3.2. Analysis of Strength Properties of Modified Saw Muds After Dry and Wet Cycle Action

#### 3.2.1. Strength Effect of Fly Ash Dosage After Dry and Wet Cycling

**Figure 8:**
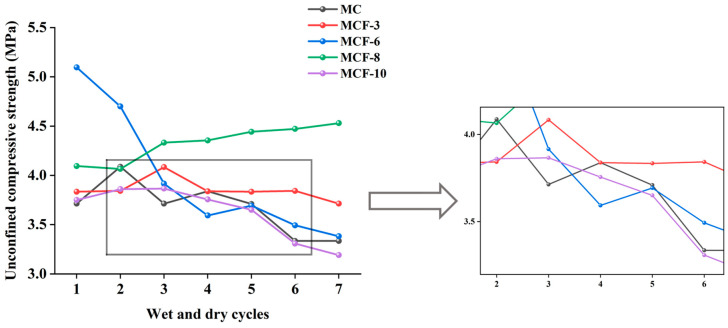
Comparison of strengths of modified saw muds with fly ash incorporation.

Figure 8 demonstrates the effects of different fly ash dosages on the strength of modified sawdusts after the action of dry and wet cycles. From the figure, it can be found that the strength of MCF-3 changed less during wet and dry cycling, showing good stability and certain wet and dry resistance. In contrast, the decrease in the MCF-6 curve was more obvious, indicating that its strength was significantly reduced after wet and dry cycling. The strength profile of MCF-8 was consistently higher than the profile of MC after wet and dry cycling and lacked a decreasing trend, which was attributed to the more adequate hydration reaction of the 8% admixture of fly ash, echoing the findings of Nayak et al. [13], who found that concrete with fly-ash admixture exhibited good durability. The curve of MCF-10 was intertwined with the curve of MC and showed a fluctuating decreasing tendency, which indicates that the strength of the modified saw mud was relatively weak in this dosage and that it had relatively weak dry and wet resistance.

#### 3.2.2. Strength Effect of Silt Admixture After Wet and Dry Cycling

**Figure 9:**
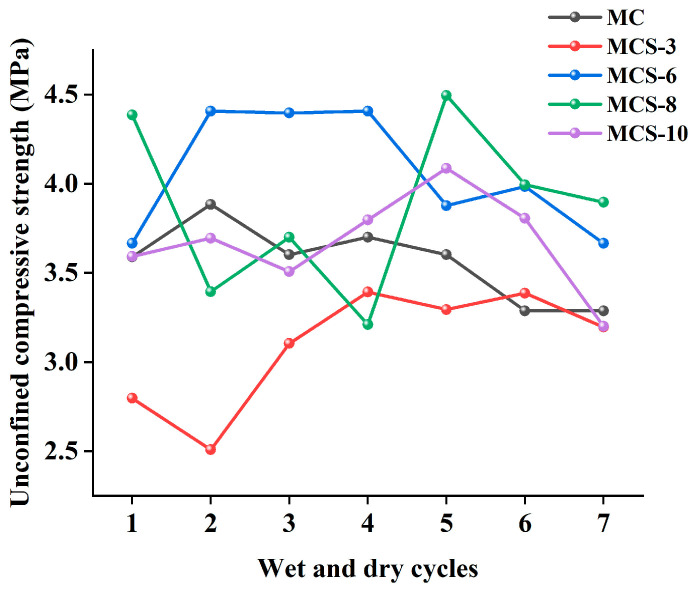
Comparison of strengths of modified saw muds with silt incorporation.

Figure 9 shows the strength comparison of modified sawdusts with different silt doping after wet and dry cycling. The strength of MCS-3 was relatively low after wet and dry cycling, but as the cycling continued, the hydration reaction was promoted, which led to an increase in its unconfined compressive strength. The curve of MCS-6 showed a trend of increasing, then stabilizing, and then decreasing and was always higher than the curve of MC, which indicates that doping of 6% silt can effectively increase the unconfined compressive strength and resistance to dryness and wetness of sawdust. The strength fluctuation of MCS-8 was more significant, showing that it was more affected by wet and dry cycling. The MCS-10 curve was intertwined with the MC curve, which shows that the 10% silt blending had limited effect on the compressive strength and wet and dry resistance of saw mud.

#### 3.2.3. Strength Effect of Red Mud Dosage After Wet and Dry Cycling

**Figure 10:**
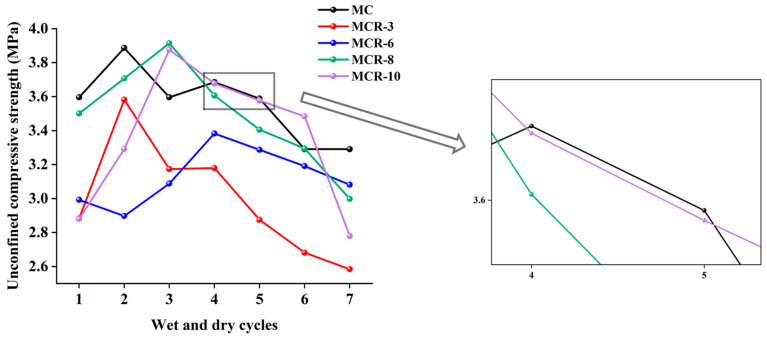
Comparison of strengths of modified saw muds mixed with red mud.

Figure 10 shows a comparison of the strengths of modified sawdusts with different red mud dosages after wet and dry cycling. From the figure, it can be seen that the curves of MCR-3 and MCR-6 were completely located below the MC curve, indicating that the strength of the modified sawdust at these two dosages was relatively low and that the wet and dry resistance was weak. This was different from the findings of Zhang et al. [23], who found that red mud had good frost resistance and durability in asphalt mixtures. This difference may be attributed to the different properties and different contents used in the two studies and to the interaction between the sawdust and red mud used in this study. The unconfined compressive strength of MCR-8 under the same number of wet and dry cycles was higher than that of other dosages, indicating that the modified sawdust at this dosage had higher wet and dry resistance.

#### 3.2.4. Comprehensive Comparison of the Strengths of Different Materials After Wet and Dry Cycling

**Figure 11:**
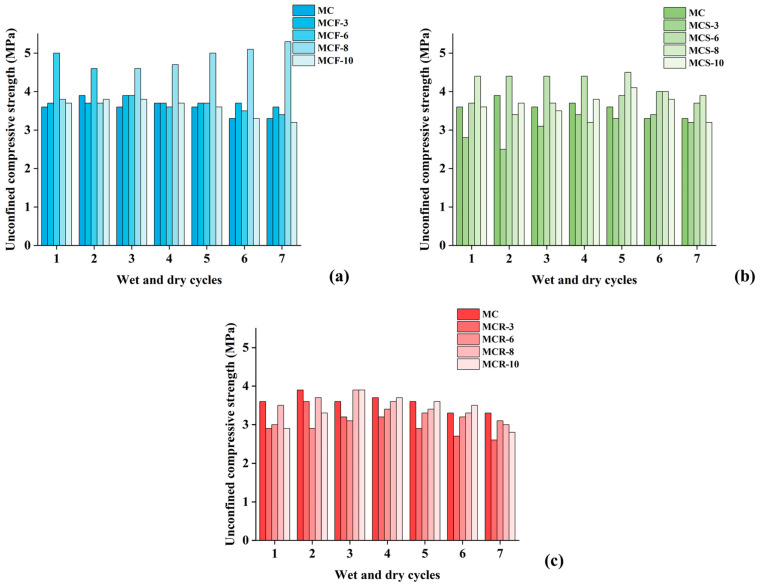
Variation in unconfined compressive strengths of modified saw muds under dry and wet cycles: (**a**) fly-ash-modified saw mud; (**b**) silt-modified saw mud; (**c**) red-mud-modified saw mud.

Figure 11 comprehensively shows the changes in the unconfined compressive strengths of different modified saw muds after dry and wet cycles. The results show that the unconfined compressive strengths of all modified saw muds remained above 2.5 MPa after seven wet and dry cycles, a strength level that meets the requirements for subbase and subgrade for highways, primary roads, secondary roads, and lower grades for light and medium traffic, as well as the strength criteria for subbase under heavy traffic conditions.

Figure 11a shows the strength variation in MCF under wet and dry cycles, from which it can be seen that the magnitudes of the unconfined compressive strengths of the specimens were 6% > 8% > 10% = 3% > 0% at the initial stage and that the magnitudes of the strengths after seven cycles were 8% > 3% > 6% > 0% > 10%. This indicates that the number of wet and dry cycles had a greater effect on the unconfined compressive strength of MCF. The strength of MCF-8 was maximized after wet and dry cycles, which indicates that the inclusion of 8% fly ash could effectively improve the resistance to wet and dry in specimens. The maximum strength loss of the composites was observed when 10% fly ash was incorporated, so excessive fly ash reduced the dry and wet resistance of modified sawdust. Figure 11b shows the strength changes in MCS under wet and dry cycles. The unconfined compressive strengths of the specimens after the first wet and dry cycle were 8% > 6% > 10% > 0% > 3%, and the strengths after the seventh cycle were 8% > 6% > 0% > 3% = 10%. Before and after the dry and wet cycles, the modified composites’ strength order was basically unchanged, but the strength was reduced, and the loss of strength was about 1 MPa, which indicates that in general, dry and wet cycles reduce the strength of such modified sawdust composites. Figure 11c shows the strength changes in MCR under wet and dry cycling, from which it can be seen that after the first wet and dry cycle, the unconfined compressive strengths of the specimens were 8% > 10% > 3% > 0% = 6%, and the strengths after the seventh cycle were 8% > 6% > 0% > 10% = 3%. Before and after the dry and wet cycles, the modified composites’ strength order changed greatly. Except for 8% and 6% doping, the strength of MCR was less than that of MC. In addition, the compressive strength of MCR was reduced more obviously compared with MCF and MCS, which indicates that red mud was less resistant to dry and wet cycling.

### 3.3. Analysis of Expansion and Contraction Characteristics of Modified Saw Muds

Expansion and contraction of soil may cause pavement bulge and collapse, resulting in uneven pavement deformation. The study of the expansion and contraction characteristics of modified sawdust provides a basis for the synergistic use of solid waste and resource utilization. According to the experimental results of the previous study, the performance of the modified sawdusts mixed with the three materials was acceptable when the solid waste dosage was 8%. Therefore, MC and MCF-8, MCS-8, and MCR-8 were selected for expansion and contraction experiments.

**Figure 12:**
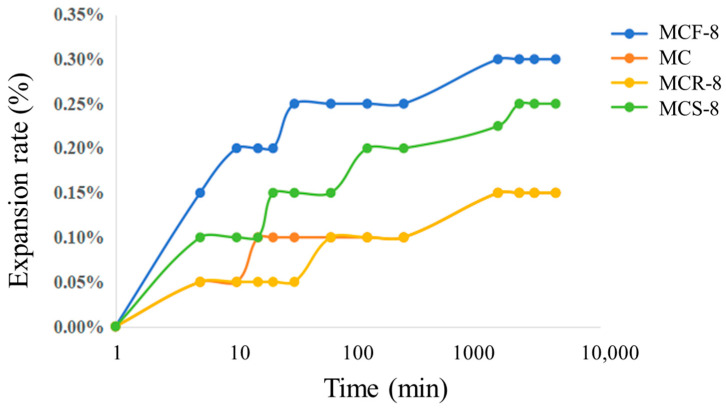
Comparison of expansion rates of modified saw mud.

The curves of the modified sawdust expansion rate with time are shown in Figure 12. Overall, most of the specimens showed rapid growth in their expansion rates at the beginning, and then the growth rates slowed down until they reached a steady state. The MC curve and the MCR-8 curve basically overlapped, but the expansion rate of MC first rose to 0.1% at about 10 min and stabilized at a later stage when it increased to 0.15%. The expansion rate of MCF-8 was the fastest in the period between 10 and 100 min, and the final expansion rate was also the highest, reaching 0.3%. The swelling of modified sawdust is mainly caused by the diffusion of soil particles, which slows down and decreases the swelling rate in the later stages of the experiment. The MCS-8 curve had more changes than the other curves. Before 10 min, the expansion rate was 0.10%; between 10 and 100 min, the expansion rate first reached 0.15 and then rose to 0.2%; and finally, after 1000 min, the expansion rate reached 0.25% and then did not grow anymore. MCR-8 had a lower expansion rate than the other modified sawdusts. Overall, the highest final expansion rate was 0.3%, and the lowest was 0.15%, less than 1%, which indicates that the modified sawdust was stable in nature and little affected by expansion due to water immersion.

**Figure 13:**
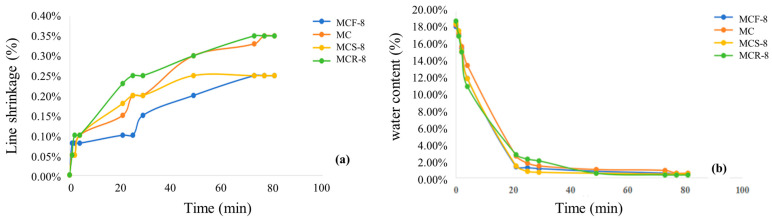
Shrinkage test results of modified saw muds: (**a**) change in shrinkage of modified saw muds; (**b**) change in water content of modified saw muds.

Figure 13 shows the line shrinkages and water content changes in modified saw muds. From the figure, it can be seen that the line shrinkage of MC was basically stable after 70 h. The change was obvious in the first 4 h; the rate of change was fast, almost a straight line, with a maximum value of 0.35%. MCF-8 line shrinkage was basically stable after 70 h. The change was obvious in the first 10 h; the rate of change was fast, and then it tended to be flat, reaching a maximum value of 0.25%, which was less than 1%. MCS-8 line shrinkage was obvious in the first 4 h; the rate of change was fast, up to 0.08% or so, in 40 h after the basic stability, reaching a maximum value of 0.25%. The MCR-8 line shrinkage rate showed the fastest change. The final shrinkage rate was also the highest, at 0.35%. For all four kinds of materials, the water content curves basically overlapped. The basic rate of reduction in the first 20 h was very fast, down to less than 2%, indicating that the specimen was basically completely dehumidified. After 20 h, the rate of change slowed to zero. Overall, the shrinkage performance of the four modified sawdusts was poor, with a maximum final shrinkage rate of 0.35% and a minimum of about 0.25%, less than 1%. The water content also basically decreased to zero after 20 h, indicating that the modified sawmill was stable in nature and very little affected by expansion due to water immersion.

### 3.4. Expansion and Contraction Characterization of Modified Saw Muds After Dry and Wet Cycling Effects

**Figure 14:**
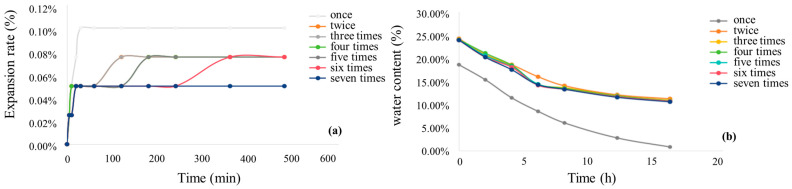

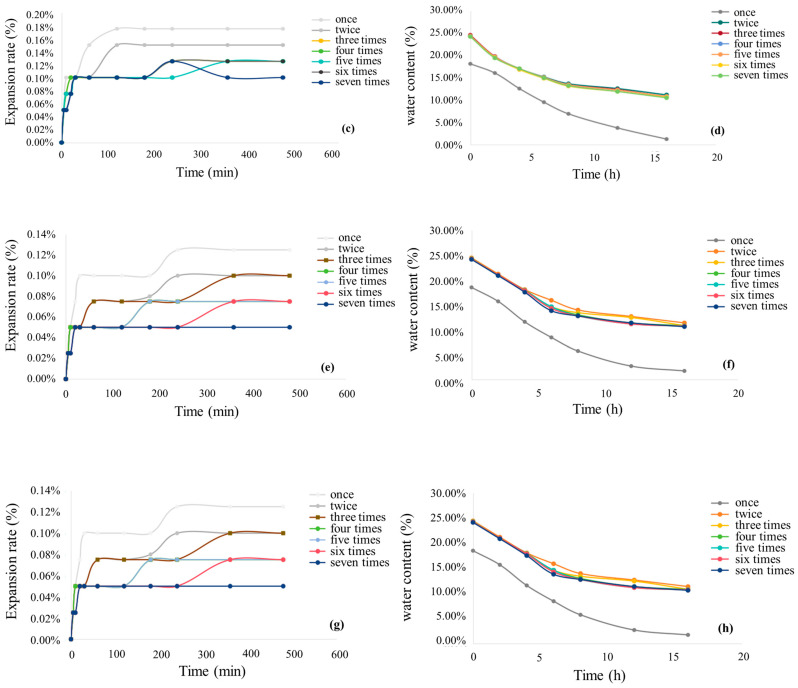
Time-dependent curves of expansion and moisture content under wet and dry cycles for modified saw mud: (**a**,**b**) cement-modified saw mud; (**c**,**d**) fly ash–cement-modified saw mud; (**e**,**f**) silt–cement-modified saw mud; (**g**,**h**) red mud–cement-modified saw mud.

Figure 14 shows the curves of expansion rate and water content with time for MC, MCF-8, MCS-8, and MCR-8 after the first through seventh wet and dry cycles. Overall, the general direction of the expansion rates of the four materials with different numbers of wet and dry cycles rapidly increased in the early stage and then stabilized over time, and the expansion rate after the first wet and dry cycle was greater than that after the second through seventh cycles. The water content of the four materials under different numbers of wet and dry cycles decreased with time, and the water content after the first wet and dry cycle was smaller than that after the second through seventh cycles. The water content curves of the second through sixth dry cycles almost overlapped, and the lowest water content was about 10%. As can be seen in Figure 14a,b, the expansion rate of MC after the first dry–wet cycle reached the highest value of 0.10% at 30 min. With increasing numbers of wet and dry cycles, the maximum value of the expansion rate gradually decreased to 0.05%. From Figure 14c,d, it can be seen that the expansion rate of MCF-8 after the first wet and dry cycle reached a maximum of 0.18% at 100 min. As the number of wet and dry cycles increased, the maximum value of the swelling rate gradually decreased to 0.1%. From Figure 14e,f, it can be seen that the expansion rate of MCS-8 after the first wet and dry cycle reached a maximum value of 0.12% at 30 min. With increasing numbers of wet and dry cycles, the maximum value of the expansion rate gradually decreased to 0.05%. From (g) and (h), it can be seen that the expansion rate of MCR-8 after the first wet and dry cycle reached 0.1% in the first 30 min and reached the maximum value of 0.12% at 200 min. With increasing numbers of wet and dry cycles, the maximum value of the expansion rate gradually decreased to 0.05%. After seven cycles, the expansion rate of the modified sawdust was less than 0.2%, and the maximum value of the expansion rate, which was above 0.04%, decreased with the number of wet and dry cycles. Therefore, the expansion rate of modified sawdust after the overall wet and dry cycles was very low and stable.

### 3.5. Analysis of SEM-EDS Test Results

#### 3.5.1. Microstructures of Modified Saw Muds

**Figure 15:**
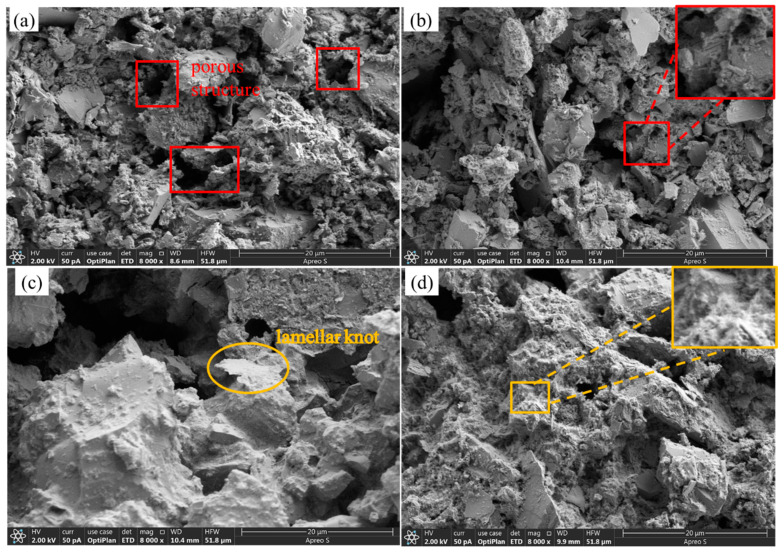
SEM images of specimens maintained for 28 d: (**a**) MC; (**b**) MCF-8; (**c**) MCS-8; (**d**) MCR-8.

Figure 15 shows the SEM micrographs of MC, MCF-8, MCS-8, and MCR-8 at 8000×. As shown in Figure 15a, it can be clearly seen that there were pores and more flocculent and gelatinous tissues in MC after 28 d of maintenance. This indicates that the compactness of the unmodified treated sawdust was low. The existence of holes provided a channel for moisture and air, which may accelerate the aging and destruction process of the material. From Figure 15b, it can be seen that there were many flocculated tissues and crystals in the MCF-8 after 28 d of conservation. There were also agglomerated tissues and some needles. The volcanic ash reaction of fly ash promotes the formation of C-S-H gels and calomelite crystals, and these crystals and flocculated tissues help to improve the strength and durability of the material. As can be seen in Figure 15c, there was a significant increase in flocculated tissues in pore sites after 28 d of maintenance of MCS-8. Flocculated tissues filled up the pores, and flakelike structures were present. As can be seen in Figure 15d, MCR-8 after 28 d of maintenance had many fewer and smaller pores relative to MC, which was due to the high alkalinity of the red mud promoting a faster hydration reaction, resulting in denser tissues, gel tissues, and crystals to fill the pores.

**Figure 16:**
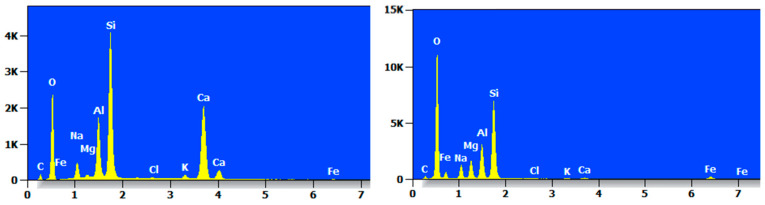
EDS diagram of MC.

Taking two reference points, the MC specimens were elementally analyzed by EDS, and their EDS patterns are shown in Figure 16, which clearly shows that the highest proportions of elements in the MC were of O and Si. The elemental content was not consistent, but the types of elements contained were basically the same, mainly O, Si, C, Na, Mg, Al, Ca, K, Fe, and so on.

**Figure 17:**
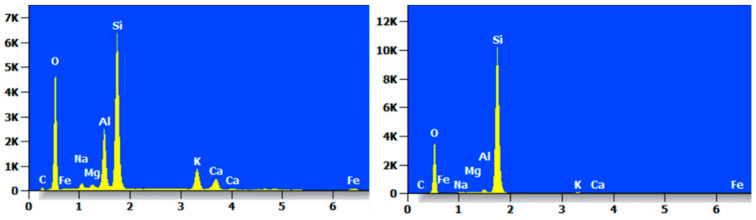
EDS diagram of MCF-8.

The EDS pattern of MCF-8 is shown in Figure 17, and it can be clearly seen that each point had a high content of O and Si elements and contained C, O, Na, Mg, Al, Si, and Ca.

**Figure 18:**
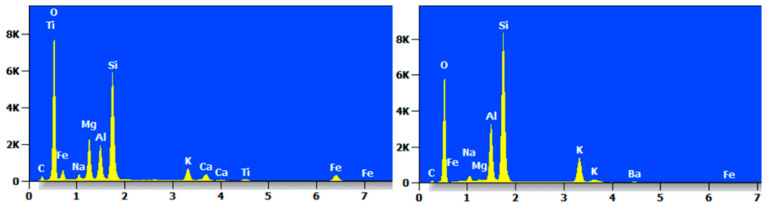
EDS diagram of MCS-8.

The EDS spectrum of MCS-8 is shown in Figure 18, which shows that the contents of O and Si were high. The MCS-8 contained mainly C, O, Na, Mg, Al, Si, K, Ca, Fe, and Ti and Ba trace elements.

**Figure 19:**
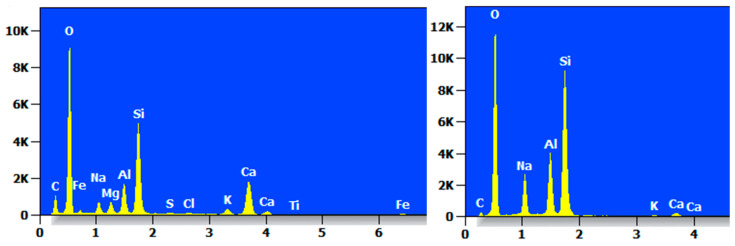
EDS diagram of MCR-8.

The EDS spectrum of MCR-8 is shown in Figure 19, from which it can be seen that the content of O and Si at each point accounted for most of the content, but there was a large difference in the types of elements between the two points, mainly C, O, Na, Al, Si, K, Ca, and so on.

#### 3.5.2. Microstructures of Modified Saw Muds After Wet and Dry Cycles

**Figure 20:**
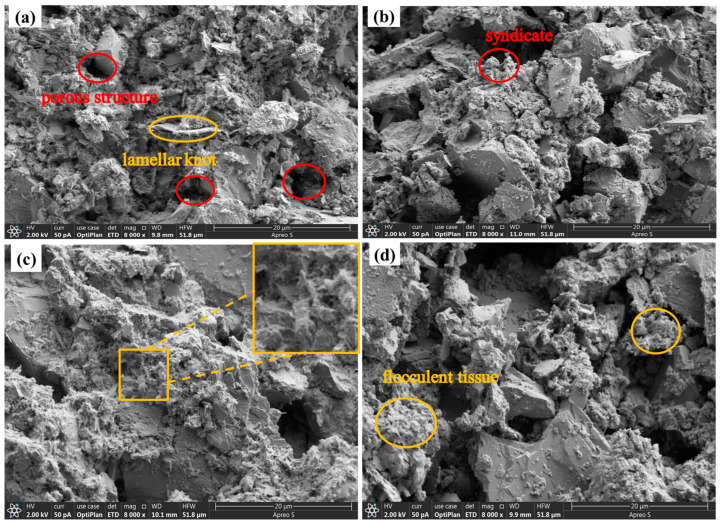
SEM images of modified saw mud specimens under dry and wet cycles: (**a**) MC; (**b**) MCF-8; (**c**) MCS-8; (**d**) MCR-8.

Figure 20 shows the SEM micrographs of MC, MCF-8, MCS-8, and MCR-8 at 8000× after seven wet and dry cycles. As shown in Figure 20a, it can be clearly seen that there were more holes in the MC after seven cycles, but there were lamellar structures and amorphous gel tissues produced, as well as some needlelike materials. This was due to the fact that the pH of the modified sawdust decreased with wet and dry cycling. Calcite exists in highly alkaline environments, and the decrease in pH led to the decomposition of calcite. During dry and wet cycling, the hydration products were destroyed, the cemented tissue flowed out, and the soil particles became loose. As shown in Figure 20b, the MCF-8 after seven dry and wet cycles had large amounts of dense flocculated tissues and crystals, and agglomerated tissues were present after the modification of the fly ash and cement ash. As shown in Figure 20c, in MCS-8 after sevenfold wet and dry cycling, many dense flocculated tissues and crystals filled the pores, and there were needlelike tissues present. The addition of silt promoted the formation of more C-S-H gels, and these gels and flocculated tissues helped to reduce the pores. As shown in Figure 20d, MCR-8, after sevenfold wet and dry cycling, had large amounts of dense flocculated tissues and crystalline structures filling the pores, and the presence of needlelike organization can be seen. After seven cycles, the crystal structure was looser compared with MCS-8.

**Figure 21:**
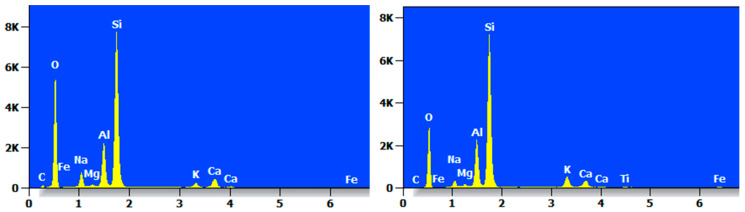
EDS diagram of MC after dry and wet cycling action.

Two reference points were taken, and the elemental analysis of the MC after seven wet and dry cycles was carried out by EDS. Its EDS spectrum is shown in Figure 21, which clearly shows that the content of O and Si elements in the two points of the specimen after sevenfold wet and dry cycling was still very high and that the MC mainly contained elements such as O, Si, Na, Mg, Al, etc., as well as the trace element Ti.

**Figure 22:**
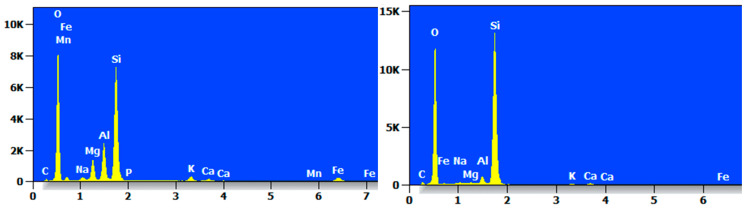
EDS diagram of MCF-8 after dry and wet cycling action.

The EDS pattern of MCF-8 after seven dry and wet cycles is shown in Figure 22, from which it can be clearly seen that the elements of O and Si accounted for the majority of the components and that other elements accounted for less.

**Figure 23:**
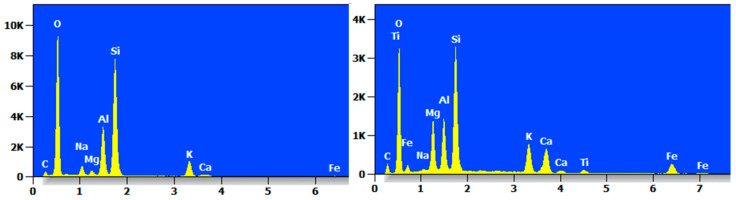
EDS diagram of MCS-8 after dry and wet cycling action.

The EDS profile of the MCS-8 after seven wet and dry cycles is shown in Figure 23. It mainly contained elements such as O, Si, Na, Mg, and Al, as well as the trace element Ti.

**Figure 24:**
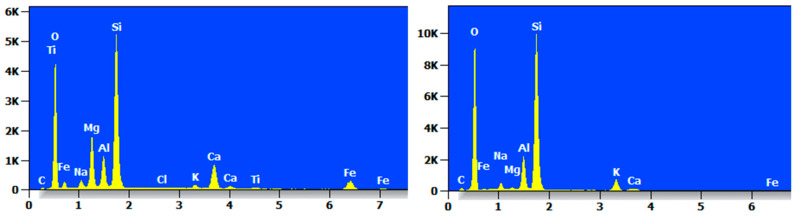
EDS diagram of MCR-8 after dry and wet cycling action.

The EDS profile of the MCR-8 after seven wet and dry cycles is shown in Figure 24, which clearly shows that the content of O and Si elements at each point was high and that the content varied greatly from point to point.

## 4. Conclusions

(1) The strength of modified sawdust after 7 days of maintenance met the requirements of the strength standard of pavement subgrade, in which the modified sawdust with 6% fly ash dosing performed the best. In addition, the strength was high when 8% solid waste was added, while 10% dosing resulted in lower strength. SEM tests showed that with increasing maintenance time, the pore space was reduced, the C-S-H gel and crystal structure increased, the strength was improved, and the needlelike organization was positively correlated with the strength.
(2) In the dry and wet cycle stage, the specimens with 8% fly ash dosing showed good resistance to dry and wet, and the strength continued to increase. The strengths of the modified sawdusts with 3% and 10% solid waste dosing was lower than that of the modified sawdust without solid waste dosing after dry and wet cycles, and the resistance to dry and wet was poor.
(3) The expansion and shrinkage of the modified saw muds were lower than 0.5%, showing good volume stability. After the first dry and wet cycle, the shrinkage of the modified sawdust decreased significantly, with little change in subsequent cycles. The expansion rate gradually decreased with increasing numbers of cycles.
(4) Under the condition that the cement doping was fixed at 6%, the modified sawdust with the MCF-6 proportion (sawdust–cement–fly ash = 88:6:6) had the best performance, and its strength was sufficient to meet the demand of road subgrade and part of the base layer. At the same time, the modified sawdust mixed with 8% solid waste (sawdust–cement–solid waste = 86:6:8) also showed good performance.
(5) The direction of future development can focus on further optimizing the amount of solid wastes and the mixing ratio in order to prepare higher-performance modified sawdust. At the same time, an in-depth study of the long-term stability of modified sawdust under different environmental conditions after solid waste incorporation will help to improve the application range and economic benefits of modified sawdust. In addition, exploring the application potential of more types of solid wastes in modified sawdust, as well as their impact on the environment, is an important part of future research. Through these studies, industrial by-products can be better utilized to promote sustainable development and provide more economical and environmentally friendly material options for road construction.

## Figures and Tables

**Table 1:** Chemical compositions of various materials.

Brochure	Chemical Composition (%)									
	Al_2_O_3_	SiO_2_	Fe_2_O_3_	SO_3_	TiO_2_	MgO	CaO	K_2_O	Na_2_O	Loss
Saw mud	14.73	70.24	0.12	0.24	/	1.13	1.05	4.52	4.21	3.76
Cement	5.12	20.12	3.62	2.38	/	2.07	63.56	/	0.53	2.6
Fly ash	29.7	51.36	5.26	/	1.13	1.39	3.18	4.52	0.34	3.12
Silt	12.26	60.99	4.22	10.43	/	1.69	1.86	1.75	2.97	3.83
Red mud	19.81	28.09	25.54	/	2.15	0.29	1.71	0.31	10.17	11.93

**Table 2:** Physical properties of cement.

Fineness 0.08/%	Specific Surface Area m^2^/kg	Densities kg/m^3^	Standard Consistency %	Stability (Boiling Method)	Solidification Time (Min)		Flexural Strength (MPa)		Compressive Strength (MPa)	
					Condensation	Congeal	3 Days	7 Days	3 Days	7 Days
0.4	345	3120	24.60	Qualified	95	156	6.3	7.8	28.0	39.5

**Table 3:** Liquid–plastic limits of each type of material.

Samples	Liquid–Plastic Limits		
	Plastic Limit/%	Liquid Limit/%	Plastic Limit Index
Saw mud	36	20.5	15.5
Fly ash	41.6	31.5	10.1
Silt	27.4	17.21	16.7
Red mud	28.2	22.5	25.7

**Table 4:** Preexperimental 7d unconfined compressive strength test results.

Dopant	Type of Admixture (%)		
	Fly Ash	Silt	Red Mud
5%	2.7	2.6	2.7
10%	4	3.1	3.2
15%	4.2	2.2	2.2
20%	5.4	2.6	2.8

**Table 5:** Formulation of modified sawdust.

	Serial Number	Cement (%)	Fly Ash (%)	Silt (%)	Red Mud (%)
Modified sawdust not mixed with solid waste	MC	6	-	-	-
Incorporation of fly ash series	MCF-3	6	3	-	-
	MCF-6	6	6	-	-
	MCF-8	6	8	-	-
	MCF-10	6	10	-	-
Mixture with silt series	MCS-3	6	-	3	-
	MCS-6	6	-	6	-
	MCS-8	6	-	8	-
	MCS-10	6	-	10	-
Mixture with red mud series	MCR-3	6	-	-	3
	MCR-6	6	-	-	6
	MCR-8	6	-	-	8
	MCR-10	6	-	-	10

## Data Availability

The data presented in this study are available on request from the corresponding author due to the data are not publicly available due to privacy or ethical restrictions.
